# Comparison of Asymptomatic Brain Lesions Between Thalassemia Major and Sickle Cell Anemia Patients

**DOI:** 10.3390/medicina61010159

**Published:** 2025-01-19

**Authors:** Derya Yavuz Demiray, Özge Eriş Davut, Gönül Oktay

**Affiliations:** 1 Department of Neurology, School of Medicine, Mustafa Kemal University, Hatay 31100, Turkey; 2 Department of Psychiatry, Ankara Etlik City Hospital, Ankara 06710, Turkey; ozgeeris@hotmail.com; 3 Department of Thalassemia Unit, Hatay Education and Research Hospital, Hatay 31027, Turkey; drgonuloktay@hotmail.com

**Keywords:** thalassemia major, sickle cell anemia, silent infarct, vascular pathology, blood levels, complications, management, neuropathic pain, depression

## Abstract

*Background and Objectives:* This study aimed to identify asymptomatic brain lesions in patients with β-thalassemia major (TM) and sickle cell anemia (SCA) and evaluate the correlation of these lesions with factors such as splenectomy, thrombocytosis, and blood transfusions. *Materials and Methods:* A total of 26 patients with thalassemia major and 23 patients with sickle cell anemia were included. Ischemic lesions were categorized as lacunar, small vessel, or multifocal. Variables including age, years of education, presence and type of MRI-detected ischemia, smoking status, hemoglobin, hematocrit, platelet count, ferritin levels, vitamin B12 levels, fasting blood sugar, splenectomy status, chelation therapy, and hydroxyurea treatment were compared between the two groups. *Results:* The mean age was 27.33 years in the thalassemia major group and 32.65 years in the sickle cell anemia group (*p* = 0.010). No statistically significant difference was observed in the distribution of ischemia types between the groups (*p* = 0.303). The thalassemia major group had a lower mean hemoglobin level (8.37 g/dL) compared to the sickle cell anemia group (9.57 g/dL) (*p* = 0.003). Ferritin levels were significantly higher in the thalassemia major group (2018.92 ng/mL) than in the sickle cell anemia group (660.39 ng/mL) (*p* < 0.001). *Conclusions:* Although ischemic lesions were more frequently observed in patients with sickle cell anemia, the difference was not statistically significant. These findings emphasize the importance of ongoing surveillance and individualized management to mitigate cerebrovascular risks in both patient populations.

## 1. Introduction

Thalassemia major (TM) and sickle cell anemia (SCA) are genetic hematological disorders that not only impair red blood cell function but also significantly impact multiple organ systems, including the central nervous system. One of the critical yet underrecognized complications in these patients is the presence of silent brain lesions or silent cerebral infarcts (SCIs), which occur without overt neurological symptoms but can lead to long-term cognitive decline, increased stroke risk, and diminished quality of life [1,2].

Silent brain lesions, detected incidentally through neuroimaging, are increasingly being acknowledged as harbingers of future ischemic events. In patients with SCA, SCIs are associated with a 14-fold higher risk of overt stroke, while in TM patients, chronic anemia and iron overload contribute to a prothrombotic state, further increasing cerebrovascular vulnerability [3,4]. Despite the absence of immediate clinical symptoms, the long-term consequences of these lesions, such as cognitive impairment, silent progression to overt stroke, and neurodegeneration, highlight the necessity for early identification and intervention [5].

Advances in medical care have significantly improved the life expectancy of patients with thalassemia and sickle cell disease (SCD). However, this has also unmasked previously underappreciated complications such as silent infarcts. Recent studies emphasize that up to 40% of patients with SCA may harbor silent brain lesions, even in the absence of clinical stroke history [6,7]. In TM, the incidence of cerebrovascular pathology is similarly rising due to iron overload and thrombophilic factors, with ischemic changes detected in nearly 60% of splenectomized patients [8].

Neuroimaging is essential in diagnosing, managing, and preventing complications associated with thalassemia and SCD. Ischemic stroke, a severe complication in SCA, is a leading cause of cognitive impairment, disability, and mortality [5,9]. These neurological complications impact the brain parenchyma, vasculature, and skull, primarily due to vasculopathy that involves both small and large blood vessels. Vaso-occlusion can lead to ischemic stroke, while ischemic damage that occurs without acute neurological symptoms and is identifiable only through neuroimaging is referred to as silent cerebral ischemia [10]. The risk of these complications is most pronounced during the first decade of life [11]. If left untreated, approximately 7.4%, 11%, and 24% of patients with SCD are expected to experience an ischemic stroke by the ages of 14, 24, and 45 years, respectively [11,12,13]. Based on the available sources, there is a need to investigate the frequency of asymptomatic brain lesions in patients with thalassemia major and sickle cell anemia and evaluate the potential risk factors associated with these lesions [6,14,15]. Some potential risk factors that have been suggested by previous research include splenectomy, thrombocytosis, blood transfusions, recipient sex, and clinical parameters such as smoking status, hemoglobin levels, hematocrit levels, platelet count, ferritin level, fasting blood sugar, and presence of complications [6,14,15,16,17].

This study aims to compare the presence of silent infarcts and variations in blood and clinical parameters between patients with TM and SCA. By understanding these differences, we can improve the management and treatment strategies for these patients.

## 2. Materials and Methods

### 2.1. Study Design and Study Population

This prospective, cross-sectional study was conducted at a single center between February 2022 and December 2022. Ethical approval was obtained from the Mustafa Kemal University Local Ethics Committee (Approval Date: 14 February 2022, Protocol Number: 07). The study was carried out in accordance with the principles outlined in the Declaration of Helsinki.

Forty-nine asymptomatic patients (26 with thalassemia major and 23 with sickle cell anemia) were enrolled in this prospective study between January 2022 and December 2022. Inclusion criteria required patients to have no history of stroke-like episodes, seizures, infectious diseases, or other conditions causing brain atrophic changes, as well as normal findings on neurological examination. Demographic and clinical data were collected, including age, education level, presence and type of magnetic resonance imaging (MRI)-detected abnormalities (ischemia), smoking status and intensity, hemoglobin, hematocrit, platelet count, ferritin levels, vitamin B12 levels, depression status, fasting blood glucose, splenectomy history, complications, chelation therapy, and hydroxyurea treatment.

Exclusion criteria included the presence of neuropathy due to anatomical pathology, lumbar disk herniation, diabetes mellitus, a history of neurotoxic drug use, and peripheral nerve involvement detected during clinical examination.

All patients underwent brain MRI, which included sagittal, axial, and coronal sequences using T1, T2, FLAIR, and gradient echo protocols. Ischemic lesions were categorized as lacunar, small vessel, or multifocal. Lesions were required to be visible on at least two planes and have a minimum diameter of 3 mm on FLAIR imaging to be included in the analysis. No contrast agents were used during the imaging process.

Prior to the examinations, all patients provided both verbal and written informed consent, which included an explanation of the procedure and its purpose. The brain MRIs were performed using a GE Healthcare Signa 1.5 Tesla scanner in the Department of Radiodiagnostics, and the findings were reviewed and interpreted by academic staff specializing in radiodiagnostics.

### 2.2. Statistical Analysis

All statistical analyses were conducted using SPSS software (Statistical Package for the Social Sciences, version 21, Chicago, IL, USA). Descriptive statistics were used to summarize the dataset, with frequencies and percentages calculated for categorical variables and means, along with standard deviations reported for continuous variables. The chi-square test was employed to examine differences between groups for categorical variables, while Fisher’s exact test was applied for smaller sample sizes to maintain statistical accuracy. When significant differences emerged from the chi-square analyses, the z-ratio test was performed to pinpoint the specific sources of variation. For continuous variables, the Spearman correlation coefficient was utilized to assess the strength and direction of associations. Normality of data distributions was assessed prior to analysis, and nonparametric tests were implemented for data that violated normality assumptions. Statistical significance was defined as a *p*-value of less than 0.05, and the results were presented with 95% confidence intervals where applicable to improve the reliability and interpretability of the findings.

## 3. Results

This study included 26 patients with thalassemia major (53.1%) and 23 patients with sickle cell anemia (46.9%), forming two comparable groups. Only a minority of patients (16.3%) reported being smokers, with 19.2% in the thalassemia major group and 13% in the sickle cell anemia group. The difference between the two groups was not statistically significant (χ^2^ = 0.342, *p* = 0.559). The patients with thalassemia major were significantly younger (27.33 ± 5.65 years) compared to those with sickle cell anemia (32.65 ± 8.46 years) (t = −2.666, *p* = 0.010). Ischemic lesions identified through MRI were present in 19.2% of the patients with thalassemia major and 30.4% of those with sickle cell anemia; however, the difference was not statistically significant (*p* = 0.363). Additionally, the distribution of ischemia types (lacunar, small vessel, and multiple) showed no significant variation between the two groups (χ^2^ = 3.639, *p* = 0.303) (Table 1).

Hemoglobin levels were significantly lower in the thalassemia major group (8.37 ± 0.79 g/dL) compared to the sickle cell anemia group (9.57 ± 1.88 g/dL) (U = 150.5, *p* = 0.003). Ferritin levels were markedly higher in the thalassemia major group, with a mean of 2018.92 ± 1827.17 ng/mL, compared to 660.39 ± 749.07 ng/mL in the sickle cell anemia group (U = 109.5, *p* < 0.001). Chelation therapy was universally applied in thalassemia major patients (100%), while only 17.4% of sickle cell anemia patients underwent this treatment, a highly significant difference (χ^2^ = 35.801, *p* < 0.001) (Table 1).

Splenectomy status showed distinct patterns between the two groups. In the thalassemia major group, 50% of the patients had undergone surgical splenectomy, while the other 50% had an intact spleen. In contrast, 60.9% of sickle cell anemia patients experienced autosplenectomy, 34.8% underwent surgical splenectomy, and only 4.3% had an intact spleen. Splenectomy status was significantly associated with the type of blood disorder (χ^2^ = 25.388, *p* < 0.001), though it was not significantly related to other clinical or demographic factors. These findings highlight significant differences between the two groups in terms of age, hemoglobin and ferritin levels, chelation therapy use, and splenectomy patterns, while also identifying trends in ischemia rates and other clinical characteristics (Table 1).

**Figure 1:**
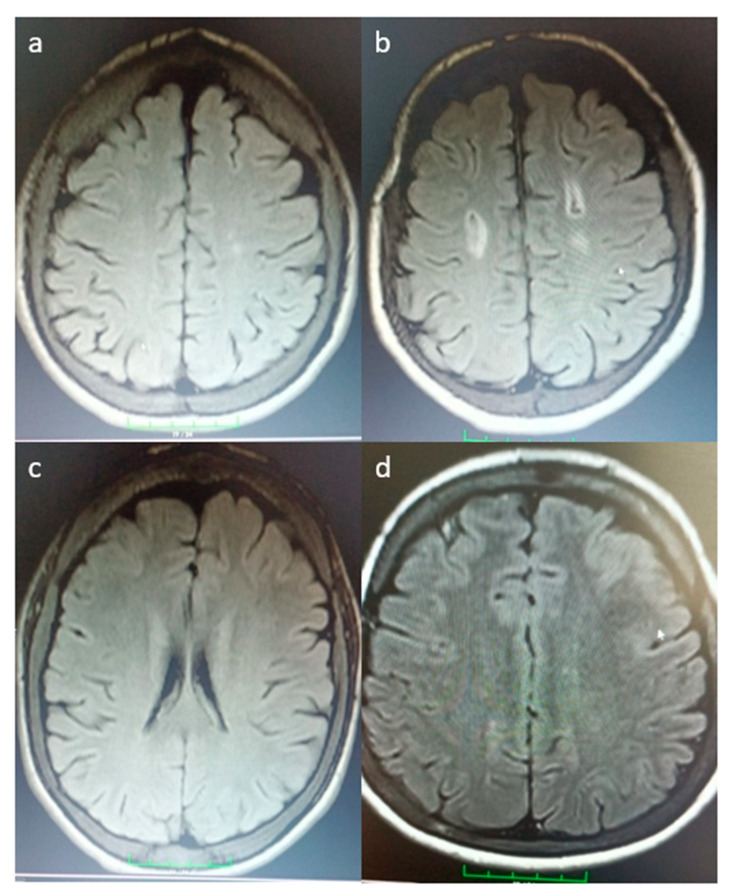
Representative MRI findings of silent cerebral infarcts in patients with thalassemia major and sickle cell anemia. (**a**) A patient with SCA showing a lacunar infarct in the deep white matter. (**b**) A patient with TM demonstrating multiple ischemic infarcts scattered throughout the periventricular and subcortical white matter, indicative of multifocal ischemic changes. (**c**) An SCA patient showing diffuse hyperintensities in the periventricular and deep white matter, representing small vessel disease with chronic ischemic changes. (**d**) A TM patient displaying a single lacunar infarct in the basal ganglia, appearing as a small, rounded hyperintense lesion, suggesting localized ischemia in a deep brain region.

We detected silent cerebral infarcts (SCIs) in 30.4% of the cases, with varying patterns observed between patients with sickle cell anemia and those with thalassemia major. Figure 1 illustrates the representative MRI findings. In a patient with sickle cell anemia, a lacunar infarct is demonstrated as a small, rounded hyperintense lesion on axial FLAIR imaging (Figure 1a). In contrast, a patient with thalassemia major exhibited multiple ischemic infarcts, appearing as scattered hyperintense regions in the white matter, indicative of multifocal ischemic changes (Figure 1b). Additionally, findings of small vessel disease were noted in a patient with sickle cell anemia, characterized by diffuse hyperintensities in the periventricular and deep white matter (Figure 1c). Lastly, a lacunar infarct was also identified in a patient with thalassemia major, presenting as a small focal hyperintense area consistent with localized ischemia (Figure 1d). These MRI findings highlight the distinct cerebrovascular manifestations in these two patient groups (Figure 1).

## 4. Discussion

This study focused on investigating the differences in asymptomatic brain lesions and blood parameters between individuals with TM and those with SCA. The results demonstrated distinct clinical and laboratory characteristics in these two groups, highlighting the unique pathophysiological processes underlying each condition. These differences are particularly significant, as they provide a deeper understanding of the neurological and hematological complications associated with TM and SCA, which could guide more tailored approaches to patient management. By identifying these disparities, the study underscores the importance of individualized diagnostic and treatment strategies, aiming to address the specific needs of each patient population and improve long-term outcomes.

In our study, no statistically significant difference was observed in the distribution of ischemia types between the groups; however, multifocal ischemic lesions were more commonly seen in the SCA group. This observation aligns with the pathophysiology of SCA, where recurrent vaso-occlusive events and chronic endothelial damage contribute to widespread cerebral ischemia. Multifocal lesions often reflect ongoing subclinical ischemic injury rather than isolated events, suggesting a more diffuse cerebrovascular involvement in SCA patients. Clinically, the presence of multifocal ischemic lesions may indicate a higher risk of progressive cognitive decline, overt stroke, and worsening cerebrovascular reserve in these patients. Studies have demonstrated that SCIs in adults with SCA are not isolated phenomena but part of a continuum that can predispose individuals to future infarct recurrence, similar to patterns seen in pediatric populations [18]. Both stroke and SCI are leading causes of brain injury in SCD, with stroke significantly contributing to morbidity and mortality [19,20]. The association between SCI and a 14-fold increased risk of overt ischemic stroke further underscores the clinical importance of detecting and monitoring these lesions [21]. Understanding the prevalence and characteristics of multifocal lesions in SCA patients can guide early intervention strategies, including the use of hydroxyurea, transfusion therapy, and regular neuroimaging surveillance. This proactive approach may help mitigate long-term neurological complications and improve overall patient outcomes.

Chronic transfusion therapy has been shown to reduce the risk of stroke by up to 92% in patients with elevated transcranial Doppler (TCD) flow velocities (≥200 cm/s) [22]. The primary mechanism underlying cerebral infarction in SCD involves abnormal erythrocyte adherence to the vascular endothelium and hemolysis. This process leads to platelet aggregation, increased vasomotor tone, and ultimately thrombosis [23]. As the disease progresses, luminal narrowing occurs due to intimal proliferation involving smooth muscle cells and fibroblasts, resulting in occlusive vasculopathy. Reduced arterial oxygen content and chronic inflammation also contribute to cerebral infarction [24]. Sickle vasculopathy is strongly linked to intravascular hemolysis, with elevated lactate dehydrogenase (LDH)—a marker of hemolysis—being associated with an increased risk of cerebrovascular complications [25]. Hemolysis also reduces bioavailable nitric oxide, promoting endothelial dysfunction and chronic vascular damage. Intimal proliferation is thought to play a pivotal role in thrombosis formation [26].

Silent infarcts in SCD typically occur in the border-zone regions and are associated with reduced cerebral blood flow [18]. Studies have shown that red blood cell transfusions can significantly reduce the risk of new SCI lesions and overt strokes, with a reported risk reduction of more than half over a 3-year follow-up period [5]. While abnormal TCD velocities serve as reliable predictors of first stroke in children with SCA, these findings are rare in adults, and the clinical utility of TCD for predicting stroke risk in adult populations remains unclear [5,19].

The prevalence of SCI is estimated to affect up to 40% of SCD patients who exhibit no clinical symptoms, including those with sickle β-thalassemia. This rate increases to over 50% in adults, with a median age of 30 years (interquartile range: 22–35 years) [5,18]. In a study by Sayman et al., the rate of SCI was reported as 22% in a cohort of SCA patients comprising 3 children, 29 adolescents, and 17 young adults [7]. In our study, SCI was detected in 30.4% of the patients, underscoring the importance of neuroimaging for early identification of asymptomatic cerebrovascular pathology in this population.

Thalassemia creates a prothrombotic state, increasing the risk of cerebrovascular events (CVEs). The combination of chronic anemia, hemolysis, ineffective erythropoiesis, and iron overload plays a significant role in the pathophysiology of this condition. Platelets in patients with thalassemia are in a persistently activated state, exhibiting enhanced aggregation and a shortened lifespan due to increased consumption. Additionally, red blood cells (RBCs) undergo oxidative damage and deformation, leading to premature destruction, aggregation, and cohesion [27]. The hypercoagulable state in thalassemia is further exacerbated by reduced levels of natural anticoagulants, such as protein C and protein S, which contribute to thrombotic events [28]. Several independent risk factors have been identified for the development of thromboembolic events (TEEs) in thalassemia syndromes. These include a history of splenectomy, serum ferritin levels exceeding 1000 ng/mL, hemoglobin levels below 9 g/dL, and an age greater than 35 years [29]. These findings highlight the multifactorial nature of thrombosis in thalassemia, emphasizing the importance of regular monitoring and tailored therapeutic interventions to mitigate these risks.

In our study, the significantly lower hemoglobin levels in TM patients compared to SCA patients reflect the chronic anemia characteristic of TM. Regular blood transfusions in TM patients aim to maintain hemoglobin levels, but the underlying ineffective erythropoiesis continues to contribute to low hemoglobin levels [30]. In contrast, SCA patients often have higher hemoglobin levels due to the presence of dense cells that are less prone to hemolysis [31]. Ferritin levels are notably higher in the TM patients. This is consistent with the chronic iron overload resulting from frequent blood transfusions [32]. Despite chelation therapy, maintaining optimal ferritin levels remains a challenge in TM patients. Elevated ferritin levels are a marker of iron overload, which can lead to various complications, including liver and endocrine dysfunction, and potentially contribute to vascular damage [33]. In our study, the thalassemia group had statistically significantly lower hemoglobin levels and higher ferritin levels compared to the SCA group.

The role of splenectomy and its correlation with thromboembolic events were examined. Splenectomy is common in TM patients due to hypersplenism, which leads to increased red cell destruction and exacerbates anemia. Post-splenectomy, these patients often exhibit thrombocytosis, a known risk factor for thromboembolic events [34]. In SCA patients, the spleen typically undergoes auto-infarction early in life, and these patients may have a different risk profile for thrombotic events compared to TM patients. In our study, 60.9% of the patients had autosplenectomy history. Splenectomy should be carefully considered only when it is expected to significantly alleviate anemia, reduce extramedullary hematopoiesis, and address growth failure. However, the procedure carries notable risks beyond post-splenectomy sepsis. Numerous studies have reported an increased incidence of complications following splenectomy in patients with thalassemia, including pulmonary hypertension, heart failure, thrombosis, cholelithiasis, leg ulcers, osteoporosis, and brain infarcts, among others [35,36]. Over the past five decades, the life expectancy of patients with thalassemia has improved considerably. This remarkable progress is largely attributable to advancements in early disease diagnosis, the implementation of intensified transfusion protocols (e.g., hyper-transfusion), and the early initiation and intensification of chelation therapy, particularly during the first decade of life. Additionally, efforts to prevent or effectively manage comorbid infections, such as hepatitis C (HCV) and HIV, have significantly contributed to better survival outcomes [37].

In thalassemia, increased platelet activation and aggregation have been well documented, alongside elevated levels of platelets expressing activation markers such as CD62P (P-selectin) and CD63. Platelet survival is notably shortened due to heightened platelet consumption, a phenomenon that is particularly pronounced in splenectomized patients. This hyperactive platelet state is a key contributor to the prothrombotic tendencies observed in individuals with thalassemia [38]. In our study, splenectomized patients demonstrated significantly higher platelet counts compared to those who had not undergone splenectomy (*p* < 0.03). This finding highlights the impact of splenectomy on platelet levels, likely due to the reduced clearance of circulating platelets in the absence of the spleen. The elevated platelet counts in splenectomized individuals may further exacerbate the hypercoagulable state, increasing the risk of thrombotic complications.

A brain MRI study on adult, splenectomized thalassemia intermedia patients showed a rate of silent white matter lesions as high as 60% [8]. In our study, in thalassemia major patients, no statistically significant difference was found between those with and without splenectomy in ischemia.

Blood transfusions are a cornerstone of TM management, helping to suppress ineffective erythropoiesis and minimize bone marrow expansion [39]. Previous studies have shown that factors such as iron overload and post-splenectomy thrombocytosis contribute to cerebrovascular complications in TM patients [3,4]. Hashemieh et al. reported a 60% prevalence of ischemic brain lesions in splenectomized TM patients [4]. This figure is higher than the 19.2% observed in our study, potentially reflecting differences in patient populations and MRI protocols. In SCA patients, a study by Sayman et al. reported a 22% prevalence of SCI [7], closely aligning with the 30.4% observed in our study. These findings reinforce the notion that silent infarcts are a common complication in SCA, even in the absence of overt neurological symptoms. DeBaun et al. demonstrated that chronic transfusion therapy reduced the risk of SCI by 58%, highlighting the protective role of transfusions in SCA management [5]. In our study, 100% of the TM patients were on chelation therapy, reflecting the need to manage iron overload due to frequent transfusions. However, only 17.4% of SCA patients required chelation therapy, suggesting a lower transfusion burden in this group.

Chelation therapy is a cornerstone of TM management to mitigate iron overload, while hydroxyurea is widely used in SCA to reduce vaso-occlusive crises and induce fetal hemoglobin production. In our study, 87% of SCA patients received hydroxyurea, while no TM patients received this treatment. Hydroxyurea has been shown to decrease the frequency of cerebrovascular events in SCA by reducing red cell sickling and promoting nitric oxide production [40]. A study by Rigano et al. reported that hydroxyurea significantly lowered the incidence of cerebrovascular events in SCA patients [20]. Our findings highlight distinct treatment approaches in TM and SCA, potentially influencing cerebrovascular outcomes. The widespread use of hydroxyurea in SCA patients may account for the reduced severity of cerebrovascular pathology despite the higher prevalence of SCI. In contrast, TM patients rely on transfusions and chelation therapy to mitigate iron overload, which indirectly reduces the risk of ischemic events.

Hydroxyurea is commonly used in SCA to reduce the frequency of pain crises and acute chest syndrome by inducing fetal hemoglobin production, which inhibits sickling [41]. The use of hydroxyurea was not prevalent in the TM group, as its benefits are specific to the pathophysiology of SCA. This treatment difference underscores the need for disease-specific management approaches. In situations where RBC transfusion is not feasible, hydroxyurea offers an alternative by increasing the production of fetal hemoglobin (HbF), thereby reducing the proportion of sickle hemoglobin (HbS). Hydroxyurea, which is specifically approved for the management of SCD, also plays a role in decreasing coagulation activity. It achieves this by reducing phospholipid expression on the surfaces of both RBCs and platelets and lowering RBC adhesion to thrombospondin. Additionally, hydroxyurea acts as a nitric oxide (NO) donor, further contributing to its antithrombotic effects. Another important mechanism of hydroxyurea is its ability to decrease hemostatic activation through the reduction of white blood cell counts, particularly monocytes, which express pro-inflammatory transcription factors. This reduction in inflammation and coagulation activity enhances vascular health in SCD patients [40]. Evidence from a cohort of patients without severe intracranial vasculopathy indicates that hydroxyurea is as effective as chronic transfusion therapy in reducing the risk of stroke [42]. These findings highlight hydroxyurea’s multifaceted benefits, not only as a therapeutic agent for managing SCD but also as a means of addressing coagulation abnormalities and reducing stroke risk. Further research is warranted to explore the long-term effects of hydroxyurea in different patient subgroups and to identify optimal strategies for integrating it into comprehensive SCD management protocols.

The distinct differences in brain MRI findings and blood parameters between TM and SCA patients suggest the necessity for tailored clinical management strategies. For TM patients, regular monitoring of iron levels and appropriate chelation therapy are crucial to preventing iron overload complications. A possible role of disease-modifying therapies, when used since childhood, is in palliating the progression of CNS disease, but not completely stopping it [43]. For SCA patients, regular screening for silent cerebral infarcts is essential.

Further research is needed to explore the mechanisms underlying these differences, particularly the role of chronic hypoxia and iron overload in the development of brain pathology in patients with TM and SCA. Chronic hypoxia, a hallmark of SCA, and iron overload, commonly observed in TM due to frequent blood transfusions, are likely to contribute significantly to the observed differences in cerebral and systemic complications. Investigating the interplay between these factors could provide a deeper understanding of their impact on asymptomatic brain lesions.

### Limitations of the Study

This study has several limitations that should be acknowledged. The small sample size may limit the generalizability of the findings, and the cross-sectional design prevents an assessment of the long-term progression of asymptomatic brain lesions and related cerebrovascular complications. Additionally, the absence of a healthy control group limits comparisons to the general population. Future research should focus on longitudinal studies to track the progression of SCI over time and evaluate their impact on cognitive function and stroke risk. Interventional trials exploring the effectiveness of early hydroxyurea use in SCA and alternative chelation therapies in TM could provide deeper insights into preventive strategies. Expanding sample sizes and incorporating diverse patient populations will further enhance the robustness and applicability of future findings.

## 5. Conclusions

In conclusion, this study highlights the prevalence of asymptomatic brain lesions in patients with TM and SCA, contributing to the understanding of cerebrovascular complications in these disorders. By identifying differences in hemoglobin, ferritin levels, and SCI rates, it underscores the distinct pathophysiological processes involved. The findings emphasize the importance of tailored treatment approaches, with chelation therapy being central to TM management and hydroxyurea being widely used in SCA. Clinically, the detection of SCI in both groups supports the need for routine neuroimaging and early intervention to reduce future stroke risk. This study reinforces the value of individualized care and calls for further research to guide preventive strategies and improve long-term neurological outcomes in these patient populations.

## Figures and Tables

**Table 1:** Descriptive statistics of socio-demographic information of patients.

Variable	Thalassemia Major (n = 26)	Sickle Cell Anemia (n = 23)	Statistics
	**Mean ± Standard Deviation (SD)**		
Age	27.33 ± 5.65	32.65 ± 8.46	t = −2.666, *p* = 0.010
Gender (female/male)	14/12	11/12	χ^2^ = 0.243, *p* = 0.622
Education year	10.11 ± 3.94	11.95 ± 4.00	t = −1.619, *p* = 0.112
MRI Ischemia	5 (%19.2)	7 (%30.4)	χ^2^ = 0.828, *p* = 0.363
Ischemia Type			χ^2^ = 3.639, *p* = 0.303
No ischemia	21 (%80.8)	16 (%69.6)	
Lacunar	3 (%11.5)	1 (%4.3)	
Small vessel	1 (%3.8)	2 (%8.7)	
Multiple	1 (%3.8)	4 (%17.4)	
Cigarette	5 (%19.2)	3 (%13.0)	χ^2^ = 0.342, *p* = 0.559
Hemoglobin (mg/dL)	8.37 ± 0.79	9.57 ± 1.88	U = 150.5, *p* = 0.003
Hematocrit	25.63 ± 1.99	27.51 ± 5.31	U = 207.5, *p* = 0.106
Platelet (×10^3^/μL)	465.36 ± 265.73	434.30 ± 170.33	U = 297.5, *p* = 0.436
Ferritin	2018.92 ± 1827.17	660.39 ± 749.07	U = 109.5, *p* < 0.001
Fasting blood glucose	100.15 ± 14.59	97.34 ± 10.31	U = 267.0, *p* = 0.528
Splenectomy			χ^2^ = 25.388, *p* < 0.001
No	13 (%50)	1 (%4.3)	
Splenectomy	13 (%50)	8 (%34.8)	
Autosplenectomy	0	14 (%60.9)	
Chelation therapy	26 (%100)	4 (%17.4)	χ^2^ = 35.801, *p* < 0.001
Hydroxyurea therapy	0	20 (%87.0)	χ^2^ = 38.201, *p* < 0.001

## Data Availability

The original contributions presented in the study are included in the article, further inquiries can be directed to the corresponding authors.

## References

[1] Chonat S., Quinn C.T. (2017). Current Standards of Care and Long Term Outcomes for Thalassemia and Sickle Cell Disease. Adv. Exp. Med. Biol..

[2] Manfre L., Giarratano E., Maggio A., Banco A., Vaccaro G., Lagalla R. (1999). MR imaging of the brain: Findings in asymptomatic patients with thalassemia intermedia and sickle cell-thalassemia disease. AJR Am. J. Roentgenol..

[3] Zafeiriou D.I., Economou M., Athanasiou-Metaxa M. (2006). Neurological complications in beta-thalassemia. Brain Dev..

[4] Hashemieh M., Jafari N. (2022). Vascular Brain Damage in Thalassemia Syndrome: An Emerging Challenge. Iran. J. Child. Neurol..

[5] DeBaun M.R., Gordon M., McKinstry R.C., Noetzel M.J., White D.A., Sarnaik S.A., Meier E.R., Howard T.H., Majumdar S., Inusa B.P. (2014). Controlled trial of transfusions for silent cerebral infarcts in sickle cell anemia. N. Engl. J. Med..

[6] Kinney T.R., Sleeper L.A., Wang W.C., Zimmerman R.A., Pegelow C.H., Ohene-Frempong K., Wethers D.L., Bello J.A., Vichinsky E.P., Moser F.G. (1999). Silent cerebral infarcts in sickle cell anemia: A risk factor analysis. The Cooperative Study of Sickle Cell Disease. Pediatrics.

[7] Sayman E., LeblebIsatan G., Leblebisatan S., Bicakci Y.K., Kilinc Y., Barutcu A. (2020). Silent cerebral infarct in sickle cell anemia patients of southern Turkey. Turk. J. Med. Sci..

[8] Genc B., Aslan K., Atay M.H., Akan H. (2024). Evaluation of microstructural changes in the brain in transfusion dependent thalassemia patients with advanced magnetic resonance imaging techniques. Neuroradiology.

[9] Hsu P., Gay J.C., Lin C.J., Rodeghier M., DeBaun M.R., Cronin R.M. (2021). Economic evaluation of regular transfusions for cerebral infarct recurrence in the Silent Cerebral Infarct Transfusion Trial. Blood Adv..

[10] Mallon D., Doig D., Dixon L., Gontsarova A., Jan W., Tona F. (2020). Neuroimaging in Sickle Cell Disease: A Review. J. Neuroimaging.

[11] Ohene-Frempong K., Weiner S.J., Sleeper L.A., Miller S.T., Embury S., Moohr J.W., Wethers D.L., Pegelow C.H., Gill F.M. (1998). Cerebrovascular accidents in sickle cell disease: Rates and risk factors. Blood.

[12] Balkaran B., Char G., Morris J.S., Thomas P.W., Serjeant B.E., Serjeant G.R. (1992). Stroke in a cohort of patients with homozygous sickle cell disease. J. Pediatr..

[13] Sonenklar M., Marks S., Donald C., Valrie C., Smith W., Sisler I. (2024). Association of Unmet Social Needs With Disease-Related Outcomes in Pediatric Patients With Sickle Cell Disease. Pediatr. Blood Cancer.

[14] Rashid N.W., Al-Allawi N., Tahir H.I. (2023). Silent Cerebral Infarcts in Iraqi Patients with Sickle Cell Disease. Hemoglobin.

[15] Kija E.N., Saunders D.E., Munubhi E., Darekar A., Barker S., Cox T.C.S., Mango M., Soka D., Komba J., Nkya D.A. (2019). Transcranial Doppler and Magnetic Resonance in Tanzanian Children With Sickle Cell Disease. Stroke.

[16] Alshalani A., Li W., Juffermans N.P., Seghatchian J., Acker J.P. (2019). Biological mechanisms implicated in adverse outcomes of sex mismatched transfusions. Transfus. Apher. Sci..

[17] Hawkins W.W., Speck E., Leonard V.G. (1954). Variation of the hemoglobin level with age and sex. Blood.

[18] Ford A.L., Ragan D.K., Fellah S., Binkley M.M., Fields M.E., Guilliams K.P., An H., Jordan L.C., McKinstry R.C., Lee J.M. (2018). Silent infarcts in sickle cell disease occur in the border zone region and are associated with low cerebral blood flow. Blood.

[19] Kwiatkowski J.L., Voeks J.H., Kanter J., Fullerton H.J., Debenham E., Brown L., Adams R.J., Post S.S.G. (2019). Ischemic stroke in children and young adults with sickle cell disease in the post-STOP era. Am. J. Hematol..

[20] Rigano P., Pecoraro A., Calvaruso G., Steinberg M.H., Iannello S., Maggio A. (2013). Cerebrovascular events in sickle cell-beta thalassemia treated with hydroxyurea: A single center prospective survey in adult Italians. Am. J. Hematol..

[21] Jones R.S., Ford A.L., Donahue M.J., Fellah S., Davis L.T., Pruthi S., Balamurugan C., Cohen R., Davis S., Debaun M.R. (2024). Distribution of Silent Cerebral Infarcts in Adults With Sickle Cell Disease. Neurology.

[22] Ferro J.M., Infante J. (2021). Cerebrovascular manifestations in hematological diseases: An update. J. Neurol..

[23] Prussien K.V., Jordan L.C., DeBaun M.R., Compas B.E. (2019). Cognitive Function in Sickle Cell Disease Across Domains, Cerebral Infarct Status, and the Lifespan: A Meta-Analysis. J. Pediatr. Psychol..

[24] Bou-Fakhredin R., Cappellini M.D., Taher A.T., De Franceschi L. (2025). Hypercoagulability in hemoglobinopathies: Decoding the thrombotic threat. Am. J. Hematol..

[25] Kato G.J., Gladwin M.T., Steinberg M.H. (2007). Deconstructing sickle cell disease: Reappraisal of the role of hemolysis in the development of clinical subphenotypes. Blood Rev..

[26] Runge A., Brazel D., Pakbaz Z. (2022). Stroke in sickle cell disease and the promise of recent disease modifying agents. J. Neurol. Sci..

[27] Kaiafa G., Savopoulos C., Kanellos I., Mylonas K.S., Tsikalakis G., Tegos T., Kakaletsis N., Hatzitolios A.I. (2017). Anemia and stroke: Where do we stand?. Acta Neurol. Scand..

[28] Odame I., Bazuaye G.N. (2024). Transfusions, disease-modifying treatments, and curative therapies for sickle cell anemia in Africa: Where are we now?. Hematology.

[29] Sleiman J., Tarhini A., Bou-Fakhredin R., Saliba A.N., Cappellini M.D., Taher A.T. (2018). Non-Transfusion-Dependent Thalassemia: An Update on Complications and Management. Int. J. Mol. Sci..

[30] Farmakis D., Porter J., Taher A., Domenica Cappellini M., Angastiniotis M., Eleftheriou A. (2022). 2021 Thalassaemia International Federation Guidelines for the Management of Transfusion-dependent Thalassemia. Hemasphere.

[31] Brousse V., El Hoss S., Isnard P., Laurance S., Lambert C., Ali L., Bonnard A., Capito C., Sarnacki S., Berrebi D. (2024). Comparative histological analysis of spleens in pediatric patients with hemolytic anemias: Insights into the pathophysiological mechanisms of spleen destruction in sickle cell anemia. Am. J. Hematol..

[32] Soliman A.T., De Sanctis V., Yassin M., Alshurafa A., Ata F., Nashwan A. (2022). Blood transfusion and iron overload in patients with Sickle Cell Disease (SCD): Personal experience and a short update of diabetes mellitus occurrence. Acta Biomed..

[33] Yang W.P., Chang H.H., Li H.Y., Lai Y.C., Huang T.Y., Tsai K.S., Lin K.H., Lin D.T., Jou S.T., Lu M.Y. (2020). Iron Overload Associated Endocrine Dysfunction Leading to Lower Bone Mineral Density in Thalassemia Major. J. Clin. Endocrinol. Metab..

[34] Abduljalil M., Saunders J., Doherty D., Dicks M., Maher C., Mehigan B., Flavin R., Flynn C.M. (2021). Evaluation of the risk factors for venous thromboembolism post splenectomy—A ten year retrospective cohort study in St James’s hospital. Ann. Med. Surg..

[35] Dobie G. (2023). Sickle Cell Disease and Thromboembolism: New Insights on the Pathophysiology, Diagnosis, and Treatment. Clin. Lab..

[36] Akca T., Ozdemir G.N., Aycicek A., Ozkaya G. (2023). Long-term Results of Splenectomy in Transfusion-dependent Thalassemia. J. Pediatr. Hematol. Oncol..

[37] Ehsan H., Wahab A., Anwer F., Iftikhar R., Yousaf M.N. (2020). Prevalence of Transfusion Transmissible Infections in Beta-Thalassemia Major Patients in Pakistan: A Systematic Review. Cureus.

[38] Haghpanah S., Hosseini-Bensenjan M., Sayadi M., Karimi M., Ramzi M., Movahed H., Eghtedari M. (2023). Risk Factors for the Occurrence of Asymptomatic Brain Lesions in Patients with beta-Thalassemia: A Systematic Review and Meta-Analysis. Clin. Lab..

[39] Porter J. (2018). Beyond transfusion therapy: New therapies in thalassemia including drugs, alternate donor transplant, and gene therapy. Hematology Am. Soc. Hematol. Educ. Program..

[40] Bou-Fakhredin R., Rivella S., Cappellini M.D., Taher A.T. (2023). Pathogenic Mechanisms in Thalassemia I: Ineffective Erythropoiesis and Hypercoagulability. Hematol. Oncol. Clin. North. Am..

[41] Reddy P.S., Cai S.W., Barrera L., King K., Badawy S.M. (2022). Higher hydroxyurea adherence among young adults with sickle cell disease compared to children and adolescents. Ann. Med..

[42] Ware R.E., de Montalembert M., Tshilolo L., Abboud M.R. (2017). Sickle cell disease. Lancet.

[43] Champlin G., Hwang S.N., Heitzer A., Ding J., Jacola L., Estepp J.H., Wang W., Ataga K.I., Owens C.L., Newman J. (2021). Progression of central nervous system disease from pediatric to young adulthood in sickle cell anemia. Exp. Biol. Med. (Maywood).

